# Evidence of Infection by H5N2 Highly Pathogenic Avian Influenza Viruses in Healthy Wild Waterfowl

**DOI:** 10.1371/journal.ppat.1000127

**Published:** 2008-08-15

**Authors:** Nicolas Gaidet, Giovanni Cattoli, Saliha Hammoumi, Scott H. Newman, Ward Hagemeijer, John Y. Takekawa, Julien Cappelle, Tim Dodman, Tony Joannis, Patricia Gil, Isabella Monne, Alice Fusaro, Ilaria Capua, Shiiwuua Manu, Pierfrancesco Micheloni, Ulf Ottosson, John H. Mshelbwala, Juan Lubroth, Joseph Domenech, François Monicat

**Affiliations:** 1 Centre de Coopération Internationale en Recherche Agronomique pour le Développement, Montpellier, France; 2 Istituto Zooprofilattico Sperimentale delle Venezie, Legnaro, Italy; 3 Food and Agriculture Organization of the United Nations, Animal Production and Health Division, Rome, Italy; 4 Wetlands International, Wageningen, The Netherlands; 5 U.S. Geological Survey, Western Ecological Research Center, Vallejo California, United States of America; 6 National Veterinary Research Institute, Vom, Nigeria; 7 AP Leventis Ornithological Research Institute, Jos, Nigeria; 8 Istituto Nazionale per la Fauna Selvatica, Bologna, Italy; 9 Ottenby Bird Observatory, Kehlen, Luxembourg; 10 Federal Department of Forestry, Abuja, Nigeria; Erasmus Medical Center, Netherlands

## Abstract

The potential existence of a wild bird reservoir for highly pathogenic avian influenza (HPAI) has been recently questioned by the spread and the persisting circulation of H5N1 HPAI viruses, responsible for concurrent outbreaks in migratory and domestic birds over Asia, Europe, and Africa. During a large-scale surveillance programme over Eastern Europe, the Middle East, and Africa, we detected avian influenza viruses of H5N2 subtype with a highly pathogenic (HP) viral genotype in healthy birds of two wild waterfowl species sampled in Nigeria. We monitored the survival and regional movements of one of the infected birds through satellite telemetry, providing a rare evidence of a non-lethal natural infection by an HP viral genotype in wild birds. Phylogenetic analysis of the H5N2 viruses revealed close genetic relationships with H5 viruses of low pathogenicity circulating in Eurasian wild and domestic ducks. In addition, genetic analysis did not reveal known gallinaceous poultry adaptive mutations, suggesting that the emergence of HP strains could have taken place in either wild or domestic ducks or in non-gallinaceous species. The presence of coexisting but genetically distinguishable avian influenza viruses with an HP viral genotype in two cohabiting species of wild waterfowl, with evidence of non-lethal infection at least in one species and without evidence of prior extensive circulation of the virus in domestic poultry, suggest that some strains with a potential high pathogenicity for poultry could be maintained in a community of wild waterfowl.

## Author Summary

Until recently, the highly pathogenic avian influenza (HPAI) viruses responsible for high mortality in some domestic poultry were considered not to have a wild bird reservoir, but to emerge in domestic poultry populations from low pathogenic viruses perpetuated in wild waterbirds. The rapid spread of H5N1 HPAI virus in 2005–2006, with concurrent outbreaks reported in both domestic and wild birds over Asia, Europe, and Africa, has raised concerns about the potential role of migratory birds in the epidemiology of the HPAI infection. Wild birds were sampled in Africa and tested by molecular and virological methods in an attempt to trace the circulation of HPAI viruses. In addition, some of these wild birds were equipped with satellite transmitters to track their local and migratory movements in relation to the potential spread of avian diseases. Avian influenza viruses (H5N2) were detected in wild waterfowl in Nigeria, and were subsequently characterized as highly pathogenic by molecular sequencing (HPAI viral genotype). Movements of one infected bird tracked by satellite telemetry revealed that it survived infection by an HP viral genotype. This result constitutes a rare finding of infection by an AIV with an HPAI viral genotype in healthy wild birds.

## Introduction

Wild waterbirds are considered the natural reservoirs of low pathogenic avian influenza (LPAI) viruses [1],[2], but highly pathogenic avian influenza (HPAI) viruses responsible for high mortality in domestic birds do not have recognised wild bird reservoirs [3]. Among the 16 hemagglutinin (HA) subtypes of avian influenza viruses (AIVs) perpetuated in wild birds, where they caused unapparent or mild disease [1],[4], H5 and H7 viruses are recognised to have the potential to become highly pathogenic (HP) in poultry. HPAI viruses are generally considered to emerge from LPAI precursors once introduced and adapted to gallinaceous poultry populations, and to not occur in wild birds [5],[6]. Prior to 2002, no HPAI virus had been isolated from free-living waterbird populations, with the exception of a large mortality event in Common Terns *Sterna hirundo* in South Africa in 1961 associated with an H5N3 HPAI infection [7], and a few isolated cases in terrestrial birds associated with AIV-infected poultry flocks [6]. The ecology of HPAI viruses has changed since 2002 with the re-emergence and spread of the Asian H5N1 HPAI virus that has been responsible for mortalities in more than 75 wild bird species in 38 countries [8]. The spread of the H5N1 HPAI virus over Asia, Europe and Africa, contemporary to the isolation of the virus in dead migratory birds, questioned the potential for wild birds to perpetuate and spread HPAI viruses.

In response to the inter-continental spread of the H5N1 HPAI virus, we sampled and tested for HPAI infection in live-caught and hunted wild birds (>11,000 birds of 144 species) in 19 countries over Eastern Europe, the Middle East and Africa during 2006 [9] and 2007. In addition, we equipped some of the wild ducks (n = 45) we caught with satellite transmitters to track their local and migratory movements in relation to the potential spread of avian diseases.

During this large-scale surveillance programme, we detected the presence of AIVs with an HP viral genotype (i.e. motif at the HA cleavage site consistent with highly pathogenic avian influenza) in two wild African waterfowl species in Nigeria. One of the infected ducks had been fitted with a satellite transmitter during a concurrent satellite telemetry survey conducted in Nigeria, allowing its movements to be monitored. Here we present combined results from the molecular analysis of the viral genome and the satellite tracking survey of wild waterfowl naturally infected by HPAI viruses.

## Results

### Wild bird surveillance for HPAI

**Figure 1:**
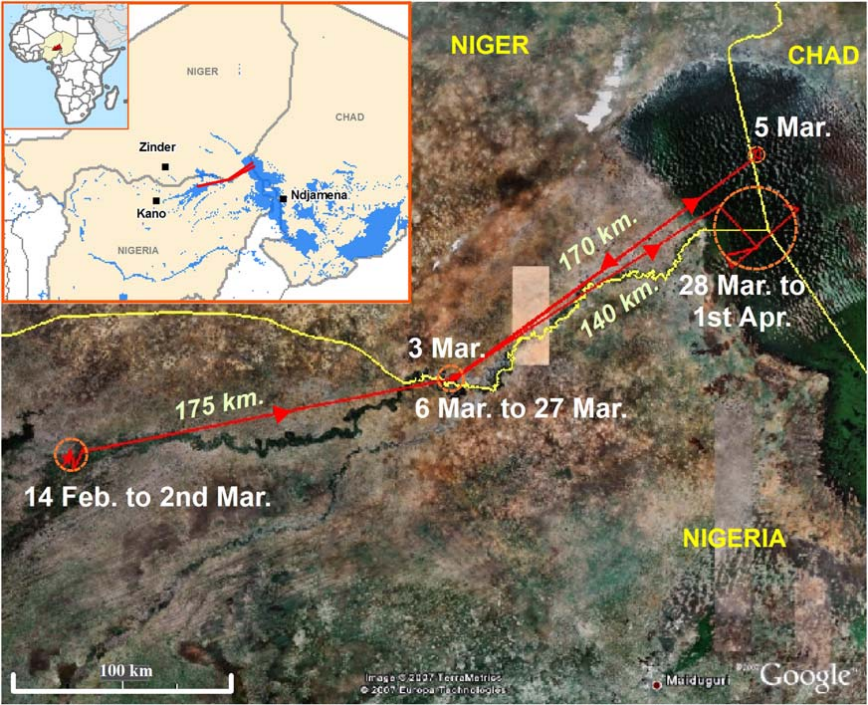
Movement paths of one White-faced Whistling Duck (*Dendrocygna viduata*) fitted with a satellite transmitter, from Hadejia-Nguru Wetlands in northern Nigeria to Lake Chad in western Chad, during February–March 2007. Dates and minimum distances between main staging areas (orange circles) are indicated on the satellite image.

AIVs of the H5N2 subtype were detected by means of molecular tests in free living and apparently healthy White-faced Whistling Duck (WFWD) *Dendrocygna viduata* and Spur-winged Goose (SWG) *Plectropterus gambensis*. These birds had been sampled at the same lake in the Hadejia-Nguru Wetlands in northern Nigeria (Jigawa State; latitude: 12°48′N; longitude: 10°44′E; Figure 1), respectively on the 14^th^ (WFWD) and 17^th^ (SWG) February 2007. In details, the oro-pharyngeal swab of one WFWD out of nine (11.1%) tested positive for type A influenza viruses and H5 subtype by RRT-PCR (Table 1). AIVs were also detected in fresh faecal samples from 8 SWG out of 97 (8.2%), of which six (6.2%) tested H5 positive. H5 positive samples showed clear positive fluorescence signal, with real time PCR Cycle threshold (Ct) values ranging from 26 to 30. Other waterbirds species (n = 122 birds), mostly waders, sampled at the same site during the same period (1–17^th^ February 2007) all tested negative (Table 1).

**Table 1:** Wild bird species sampled and tested for AIVs in Nigeria in 2007, and results of molecular tests and sequence analyses.

Bird group	Species		No. birds	No. Type A positive^a^/No. Samples			No. H5/No. Type A	HPAI^b^
				Cloaca	Oropharynx	Faeces		
Afro-tropical ducks	White-faced Whistling-Duck	*Dendrocygna viduata*	9	0/2	1/2	0/7	1/1	1
	Spur-winged Goose	*Plectropterus gambensis*	97			8/97	6/8	3
	Comb Duck	*Sarkidiornis melanotos*	8	0/7	0/8			
Eurasian duck^c^	Garganey	*Anas querquedula*	9	0/9	0/8			
Eurasian waders	Ruff	*Philomachus pugnax*	49	0/11	0/11	0/38		
	Little Stint	*Calidris minuta*	9	0/9	0/8			
	Wood Sandpiper	*Tringa glareola*	1	0/1	0/1			
	Marsh Sandpiper	*Tringa stagnatilis*	1	0/1	0/1			
	Jack Snipe	*Lymnocryptes minimus*	1	0/1	0/1			
	Black-winged Stilt^d^	*Himantopus himantopus*	1	0/1	0/1			
Afro-tropical waders	Spur-winged Lapwing	*Vanellus spinosus*	18	0/18	0/18			
	Greater Painted-Snipe	*Rostratula benghalensis*	2	0/2	0/2			
Herons	Common Squacco Heron	*Ardeola ralloides*	4	0/4	0/4			
	Yellow-billed Egret	*Egretta intermedia*	1	0/1	0/1			
	Black-crowned Night-Heron	*Nycticorax nycticorax*	1	0/1	0/1			
Passerines	Yellow Wagtail	*Motacilla flava*	16	0/16	0/16			
Raptors	Pallid Harrier	*Circus macrourus*	1	0/1	0/1			
**Total**	**17 species**		**228**	**0/85**	**1/84**	**8/142**	**7**	**4**

a Evaluated by means of real-time RT-PCR specific for type A influenza viruses (M gene).

b Evaluated by genomic sequencing.

c Birds that breed in the Western Palearctic and migrate to Africa.

d Potentially either an Afro-tropical breeding or Eurasian wintering individual.

Sequence analysis was able to reveal the presence of HPAI viral genotypes, with multiple basic amino acid motif at the cleavage site of the HA molecule (PQKEKRRKKR*GLF and PQREKRRKKR*GLF, Table 2) in four samples collected in four distinct birds (one WFWD and three SWG, Table 1). Conventional RT-PCR assay targeting the neuraminidase (NA) gene segment tested positive for the N2 subtype. No virus isolate could be obtained but sequence analysis of the entire segments encoding HA and NA proteins from PCR products confirmed the presence of AIVs of H5N2 subtype. Insufficient material of the five remaining samples was available for sequencing and further analysis.

**Table 2:** Summary of the cleavage site motif in selected African H5 HPAI and LPAI viruses.

Virus	Cleavage site	No. basic amino acids
Nigerian H5N2 HPAI	PQKEKRRKKR*GLF	7
	PQREKRRKKR*GLF	
African H5N1 HPAI 2006–2007^a^	PQGERRRKKR*GLF	6
A/chicken/Egypt/5610NAMRU3-F3/2006 (H5N1)	PQGKRRRKKR*GLF	7
A/chicken/Egypt/5611NAMRU3-AF/2006 (H5N1)	PQGKRRRKKR*GLF	7
A/Sudan/2006 (H5N1) HPAI	PQGEGRRKKR*GLF	5
A/ostrich/South Africa/N227/04 (H5N2) HPAI (9)	PQREKRRKKR*GLF	7
A/tern/South Africa/1961 (H5N3) HPAI	PQRETRRQKR*GLF	5
A/ostrich/South Africa/AI1091/2006 (HPAI)	PQRRKKR*GLF	5
A/ostrich/South Africa/AI1160/2006 (LPAI)	PQRETR*GLF	2
A/yellow-billed duck/South Africa/811/04 (H5N1LPAI)	PQRETR*GLF	2
A/mallard/Bavaria/1/2005 (H5N2 LPAI)	PQRETR*GLF	2

Cleavage site motif of the HA sequence phylogenetically most closely related to the Nigerian H5N2 HA (A/mallard/Bavaria/1/2005) is included for comparison.

a Representatives of all the sequenced African H5N1 HPAI viruses circulating in 2006–2007 (exceptions were observed in the Sudanese isolates and in 2 Egyptian isolates).

### Molecular and phylogenetic analysis

**Figure 2:**
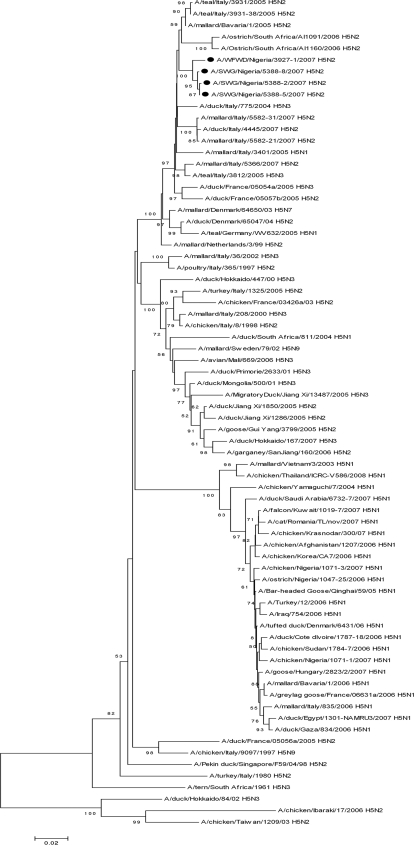
Phylogenetic tree based on the sequence analysis of the entire segment encoding for HA proteins, with representative H5N1, H5N2, and H5N3 influenza A viruses isolated in naturally infected wild and domestic birds in Asia, Europe, and Africa. Viral sequences analyzed in this study are marked with a circle.

**Figure 3:**
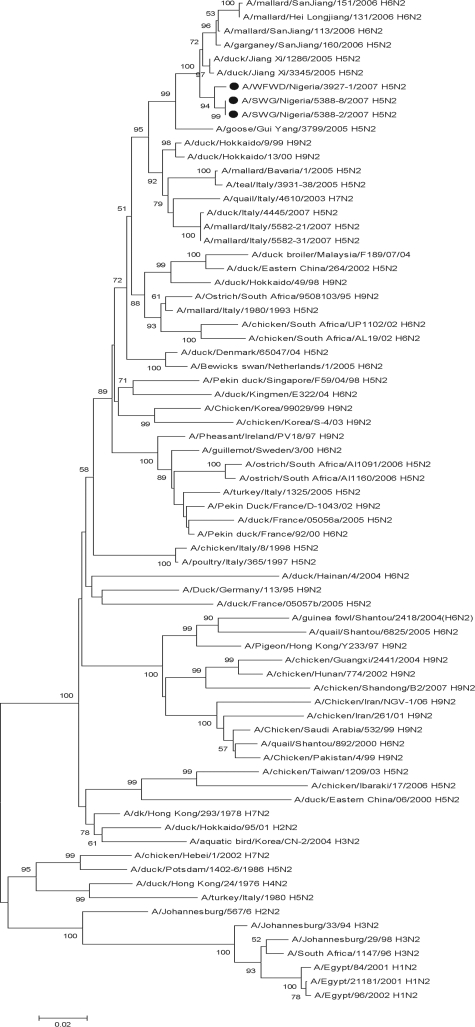
Phylogenetic tree based on the sequence analysis of the entire segment encoding for NA proteins. The phylogenetic tree includes selected N2 sequences of influenza viruses isolated in Asia, Europe and Africa. Viral sequences analyzed in this study are marked with a circle.

The site where birds were sampled is situated in Northern Nigeria where the first outbreak of H5N1 HPAI virus in Africa was reported in January 2006 [10], with many subsequent outbreaks in gallinaceous poultry or free-ranging ducks (the closest past outbreak occurred 80 km away). Unexpectedly, phylogenetic analysis of the entire HA and NA gene segments (accession numbers EU544242 to EU544248) showed that the viruses were unrelated to any strains of H5N1 HPAI viruses. Phylogenetic analysis based on the HA gene clustered the sequences with contemporary LPAI viral strains isolated in South and Central Europe and with H5 viruses isolated in South Africa (Figure 2). Nigerian H5 sequences revealed the highest homologies with the H5N2 LPAI isolate A/mallard/Bavaria/1/2005 (98.2% for A/SWG/Nigeria/5388-2-8-5/2007 and 97.9% for A/WFWD/Nigeria/3927-1/2007). Analysis of the HA deduced amino acid sequence of the Nigerian viruses A/WFWD/Nigeria/3927-1/2007 and A/SWG/Nigeria/5388-2-8-5/2007 showed high similarity with isolate A/mallard/Bavaria/1/2005, with only 11, 8 and 9 amino acid differences located outside the cleavage site, respectively. High homology at the nucleotide level, ranging from 97% to 98%, was also revealed when the sequences were compared to H5 LPAI viruses circulating in Southern Europe, A/teal/Italy/3931-38/2005 (H5N2), A/teal/Italy/3931/2005 (H5N2), A/teal/Italy/3812/2005 (H5N3) and A/mallard/Italy/5366/2007 (H5N2). Representative isolates of HPAI and LPAI H5N2 viruses recently detected in South Africa, A/ostrich/SouthAfrica/AI1091/2006 (HPAI) and A/ostrich/SouthAfrica/AI1160/2006 (LPAI), also showed high nucleotide sequence similarities (97.6%) to A/mallard/Bavaria/1/2005, but lower homologies with the Nigerian HPAI H5N2 sequences (range 95.9%–96.4%). As shown in Figure 2, A/SWG/Nigeria/5388-2-8-5/2007 and A/WFWD/Nigeria/3927-1/2007 sequences were not closely related genetically to the other H5 HPAI viruses previously detected in gallinaceous poultry in Southern Europe (e.g. A/chicken/Italy/8/98 H5N2) and in Common Tern in South Africa (A/tern/South Africa/1/61 H5N3). Phylogenetic analysis based on the NA gene revealed similarities with LPAI viruses isolated in Far East Asia (Figure 3). The highest homology (98.3%–98.5%) was observed with A/duck/Jiang Xi/1286/2005 (H5N2). The N2 sequences of A/ostrich/South Africa/AI1091/2006 (HPAI) and A/ostrich/South Africa/AI1160/2006 (LPAI) [11] were not closely related, showing homologies ranging from 89%–89.5% (Figure 3).

Genetic markers supposed to be related to gallinaceous poultry adaptation and virulence, such as potential additional glycosylation sites (AGS) in the HA molecule [12] and stalk deletion in the NA molecule [13], were not detected in the Nigerian sequences. In the NA molecule, one potential additional glycosylation site was detected in position 331 in A/WFWD/Nigeria/3927-1/2007 (S331N mutation) and in position 329 in A/SWG/Nigeria/5388-8/2007 and A/SWG/Nigeria/5388-2/2007 (D329N mutation).

Sequence analysis also revealed that two distinct H5N2 viruses with an HP viral genotype were present in these two cohabiting species of the waterfowl community. Eight and seven amino acid differences located outside the cleavage site were detected in A/WFWD/Nigeria/3927-1/2007 when compared to A/SWG/Nigeria/5388-8/2007 and A/SWG/Nigeria/5388-2/2007, A/SWG/Nigeria/5388-5/2007, respectively. NA sequence variability within the Nigerian viral genomes was less pronounced (homology 99.0%–100%). However, 5 amino acid differences were detected in A/SWG/Nigeria/5388-2-8/2007 when compared to A/WFWD/Nigeria/3927-1/2007.

### Satellite tracking

Movements of one of the H5N2 infected birds were tracked for 47 days, revealing that this bird survived infection by an HP viral genotype. This adult male WFWD had been equipped with a satellite transmitter on the date of capture and sampling, without knowing its infection status. No clinical signs were reported for this bird and it had a medium body mass respective to this species (630g [14]). It first remained for 17 days within an 8 km radius of the capture site, performing daily movement of 1–7 km (Figure 1). It then flew eastwards to Chad in four stages (140–175 km daily flights), covering a total distance of 655 km. The satellite signal disappeared a few days after this duck had settled in the interior of Lake Chad, at 360 km from the sampling site. We consider a mortality associated with HPAI infection unlikely, since the time span between capture and signal disappearance (47 days) is long, and comparatively 6 times greater than the maximum time of death which has been reported in susceptible wild waterfowl following experimental infection with H5N1 HPAI viruses (<8 days post inoculation; [15]–[18]). Signal loss, though speculative, is likely related to the death of the bird (either natural or by hunting) or to the failure of the battery of the transmitter. For comparison, another WFWD fitted with a similar transmitter in Nigeria, which tested negative for type A influenza virus (Table 1), stopped transmitting after 25 days. During this period, this non-infected WFWD (captured, sampled and equipped at the same site on the same day) had a similar movement pattern to the H5N2-infected WFWD, remaining within the same Hadejia-Nguru Wetlands area (within 17 km radius of capture site during the same first 18 day period), and performing similar short daily movements (1–18 km).

## Discussion

The H5N2 viruses we detected in two apparently healthy wild waterfowl species constitute a rare finding of infection by AIVs with an HPAI viral genotype in wild birds. Until recently, AIVs with an HPAI viral genotype had not been detected in free-living wild birds [5],[6], except for a single report of an H5N3 HPAI virus outbreak in South Africa [7], and a few cases in terrestrial birds associated with outbreaks in poultry [6],[19]. Since the emergence and spread of the Asian H5N1 virus lineage over Eurasia and Africa, H5N1 HPAI viruses have been isolated in a wide range of wild bird species. However, both these H5N3 and H5N1 HPAI viruses were isolated in sick, moribund or dead wild birds. Despite extensive global wildlife surveillance efforts, no infection with H5N1 HPAI viruses has been detected in healthy wild birds, except for a few isolated cases [20],[21]. Therefore, the significance of wild birds as a source of infection and their influence on the epidemiology of H5N1 HPAI viruses is yet to be fully established.

The two species in which these H5N2 viruses were detected are both Anatidae species, a group of wild birds with a predominant role in the perpetuation of LPAI viruses [2],[22]. Both species forage in shallow water and are highly gregarious outside the breeding season, two ecological factors associated with an increased exposure to AIV infection. The fact that we detected these H5N2 viruses at a three-day interval at the same site first suggested a virus transmission between these two cohabiting species. WFWD and SWG were the most abundant Afro-tropical ducks counted at the sampling site (53% and 40% respectively, n = 3772). Moreover, these two species, which commonly share the same habitats at feeding and roosting sites and have similar foraging behaviour and daily activity patterns [14], are prone to regular contacts or to indirect AIV transmission via shared water. However, high variation at the nucleotide and amino acid level was observed between the Nigerian H5N2 HPAI sequences detected in the WFWD and SWG. The presence of genetically distinguishable H5N2 HPAI viruses does not support the hypothesis of a cluster of infection. Rather, it indicates the co-circulation of distinct viruses in the same waterfowl community, suggesting some degree of genetic adaptation to different host species.

The survival and movements of one of the H5N2 infected duck revealed by satellite telemetry provide evidence of a non-lethal natural infection by an AIV with an HPAI viral genotype in a wild duck. This result is consistent with the absence of disease signs or mortality generally reported in ducks for most HPAI viruses. Before the Asian H5N1 HPAI viruses, no disease or mortality events associated with a natural AIV infection had been reported in either wild or domestic ducks ([5]; with the single exception of mortality cases in domestic ducks and geese during H7N1 HPAI outbreaks in Italy [23]). In addition, experimental infections with most AIV with a high pathogenicity for gallinaceous poultry (except H5N1), as well as with the A/tern/South Africa/1/61 (H5N3) [24], caused no clinical signs in domestic ducks [25] (except for A/FPV/Rostock/34 (H7N1) [26]). Lethality of HPAI viruses for duck has changed with the re-emergence of H5N1 HPAI viruses in Hong Kong in 2002 [27]–[29]. These H5N1 HPAI viruses, previously non lethal in ducks [30], have become highly lethal in some naturally infected wild ducks [27],[31], as well as in some experimentally infected wild [15],[18] and domestic ducks [28],[32]. However, H5N1 HPAI viruses show a high diversity in their pathogenicity for ducks. Results of experimental inoculations in these birds ranged from asymptomatic infection to high mortality rate, depending on species [15],[18], virus strain and age of the host (i.e. lethal only in <5-week-old domestic ducks) [33].

Telemetry results further suggest that the AIV infection did not reduce migration capacity of this duck. The movement pattern we recorded is in agreement with the described movement behaviour of WFWDs [14],[34], which are mainly sedentary but perform occasional flights of several hundred kilometres. However, the bird we tracked performed some long distance movements only 18 days after it had tested positive for HPAI virus. This period is longer than the duration of illness and viral shedding generally recorded in waterfowl inoculated with H5N1 HPAI viruses (<7 days; [15]–[18],[32]). Despite the absence of clinical symptoms at the time of capture, we cannot conclude on the ability of a wild duck to fly great distances during infection and hence to spread the virus, since no data about the persistence of viral shedding could be collected. However, the local movements (1–7 km) performed initially by this infected bird were similar in pattern and range to the movements concurrently monitored in one non-infected WFWD. Furthermore, this pattern was comparable to the short weekly distances recorded in two satellite tracked WFWD in South Africa [35], suggesting that movements were not influenced by the AIV infection.

Based on the current OIE definition [36], the molecular signature at the HA cleavage site, as revealed in the H5N2 Nigerian genomes, defines these viruses as HPAI. The acquisition of multiple basic amino acid at the HA cleavage site is recognised as a major molecular determinant of virulence in AIVs, but it might not be sufficient for the expression of high lethality [13],[37]. We did not succeed in isolating these viruses, thus *in vivo* pathogenicity tests to establish the pathogenicity for chickens [36] could not be performed. Based on the clear fluorescence signal and the low Ct values in real time PCR, the failure of the virus isolation attempt was probably not related to a sensitivity issue of the virological method. Rather, the reasons for virus isolation failure could be related to the loss of viral viability during transportation and storage or to a lack of adaptation of the viruses to the substrates used in the laboratory (i.e., SPF embryonating chicken eggs) [38]. Caution should therefore be taken to consider the phenotype of these Nigerian viruses as highly pathogenic for chickens, since few exceptions have been described involving H5 viruses with a multiple basic amino acid motif resulting in low pathogenicity in experimentally infected chickens [37],[39]. However, the number of basic amino acids at the HA cleavage site of the Nigerian H5N2 viruses was identical (n = 7) to some other African H5N1 and H5N2 HPAI viruses (Table 2). Furthermore, the cleavage site of A/SWG/Nigeria/5388-2-8-5/2007 is identical to A/ostrich/South Africa/N227/2004 (H5N2) HPAI which caused clinical signs characteristic of HPAI phenotype [11], suggesting a potential for an HPAI behaviour of these viruses.

Despite the relative proximity of our sampling site with past and recent H5N1 HPAI virus outbreaks in domestic birds in northern Nigeria (≤80 km), the H5N2 viruses we detected were unrelated to any 2004-07 H5N1 HPAI strains from Asia, Europe or Africa. The HA gene instead clustered with sequences from ducks from South and Central Europe, in particular with contemporary strains isolated from wild ducks, suggesting a connection via migratory flyways. This finding is consistent with the close relationship previously reported between H5N1 HPAI viruses isolated from Nigerian poultry and European wild birds [40] and with the role suggested for the Eurasian migratory birds in the occurrence of H5 outbreaks previously reported in Southern Africa [41],[42]. The two waterfowl species we found infected in Nigeria (i.e. WFWD and SWG) are widespread across sub-Saharan Africa, but no African populations of either species move out of the continent [14]. H5N2 viruses were detected during the mid-dry season of the Sahel zone, when large flocks of Afro-tropical duck species congregate at permanent water bodies in large floodplains. At this time of year, these birds also mix with a large number of migratory ducks originating from Eurasian breeding grounds (e.g. 50,000 Garganeys *Anas querquedula* were present on the sampling site), which gather in tropical wetlands during the northern winter. This suggests a potential role of Eurasian migratory ducks in the introduction of these viruses or their precursors into these ecosystems, which offer an interface between Eurasian and Afro-tropical wild ducks.

The proposed mechanism for the emergence of pathogenicity is that it occurs only after a virus has been introduced and adapted to gallinaceous poultry populations [5]. This theory is supported by phylogenetic analysis that demonstrated shared phylogenetic sublineages among viruses of both high and low pathogenicity [3],[43], and that identified precursors of HPAI viruses of gallinaceous poultry in wild ducks [44],[45]. However, the Nigerian HA sequences showed little or no homology with recent H5N2 or H5N1 HPAI viruses isolated from gallinaceous poultry in Europe and Africa. Further, no mutations commonly observed in gallinaceous poultry-adapted AI viruses were observed in their genomes [12],[13],[43], suggesting that these viruses may not have circulated in gallinaceous poultry, at least not in an extensive manner. This appears to be supported by the lack of evidence of a circulation in poultry of H5 viruses different from H5N1 HPAI during an extensive active surveillance conducted over Nigeria in 2007 (T. Joannis, personal communication), or in samples collected from suspected cases in Nigerian poultry in the same period H5N2 was detected in wild waterfowl (January–February 2007) [46]. The absence of molecular features associated with an extensive viral circulation in gallinaceous birds, together with the absence of evidence of circulation of H5 LPAI precursor or HPAI viruses in gallinaceous birds in Nigeria, suggests that the acquisition of an HP viral genotype could have taken place in ducks, either wild or domestic, or in other non-gallinaceous species.

The site where the birds were sampled constitutes one of the major wetlands for waterbirds in Nigeria, but is also located in one of the major areas of free-ranging duck production in this country [47]. WFWD and SWG are found in all types of natural freshwater habitats, but they also commonly feed on rice fields where domestic ducks may also feed. Local conditions are hence favourable to potential transmission events between wild and domestic ducks, through direct contact or shared water.

Though several factors contribute to virulence in AIVs, the acquisition of multiple basic amino acids at the HA cleavage site is recognised as one of the major molecular determinants in the development of HP strains [37]. The presence of coexisting but genetically distinguishable avian influenza viruses with an HP viral genotype in two cohabiting species of wild waterfowl, with evidence of non-lethal infection at least in one species and without evidence of prior extensive circulation of the virus in domestic poultry, suggest that some strains with a potential high pathogenicity for poultry could be maintained in a community of wild waterfowl.

## Methods

### Wild bird surveillance

Birds were captured using mist nets with playbacks, baited traps and nooses. Both cloacal and oropharyngeal swabs were collected from live-caught birds using sterile cotton swabs (5×15 mm and 2×10 mm size, Dutscher manufacturer). Fresh faecal samples were also collected at roosting areas (non-captured birds), ensuring that the exact species yielding the samples were identified. The health of birds was assessed by observing their movements prior to sampling, and for captured birds, by their behaviour in the hand, mass, and body condition. Samples were placed in a transport medium consisting of an isotonic phosphate buffered saline (PBS), pH 7.0–7.4, containing antibiotics and antimycotic (penicillin 10,000 units/ml, streptomycin 10 mg/ml, nystatin 1000 U/ml and gentamycin 250 µg/ml) supplemented with 20% glycerol. Samples were stored in liquid nitrogen directly in the field. An unbroken cold chain was maintained, using a liquid nitrogen container during national transport and a cryopack with dry ice during international shipment to the laboratory.

### RNA isolation, amplification, and sequence analysis

RNA was extracted at the CIRAD laboratory (Montpellier, France) with the Nucleospin RNA virus kit from Macherey Nagel using the automate Biomek FX from Beckman, after elution into 50 µl H2O. RNA was first screened for the presence of genomic nucleic acid from type A Influenza viruses by means of RRT-PCR targeting the influenza matrix gene [48]. Positive samples were tested by RT-PCR specific for H5 and H7 subtype [48],[49]. Conventional RT-PCR amplifying specifically the cleavage site was carried out on H5 or H7 positive samples and amplified products sequenced in order to detect the presence of multibasic amino acids. At the OIE/FAO Reference Laboratory for AI (IZSE, Padova, Italy), confirmatory molecular tests, including sequencing, molecular and phylogenetic analysis, were further applied and virus isolation in embryonating SPF chicken eggs was attempted for all RRT-PCR-positive samples according to standard procedures [36].

From positive samples the complete ORF of the HA and NA genomic segments were directly sequenced and phylogenetically analysed. Briefly, the cDNA of viral genomic segments 4 and 6 was amplified through the application of five to six distinct PCRs targeting overlapping regions of approximately 400 to 900 bp each. Briefly, 6 primer sets were applied to amplify and sequence 6 overlapping segments for the HA gene sequence (5′-3′ segments position −22 to 666; 422 to 1303; 900 to 1726; 557 to 1526; 729 to 1207 and 437 to 1248; referring to A/turkey/Italy/1325/05(H5N2); Genbank accession number CY022629). For the NA gene, 5 primer sets were applied to amplify and sequence five overlapping segments (5′-3′ segments position −7 to 171; 1–723; 583 to 950; 877 to 1374 and 877 to 1426; referring to A/turkey/Italy/1325/05(H5N2); Genbank accession number CY022631). Primer sequences are available on request.

Amplicons were purified (ExoSap-IT, USB Corporation, Ohio, USA) and sequenced on both strands. The obtained sequences were then aligned together with contemporary and Eurasian HPAI and LPAI sequences and their phylogenetic relationship was inferred by the application of Neighbor-Joining algorithm (MEGA 4.0; 1,000 bootstrapping) [50]. Sequences have been deposited in a public database (GenBank accession numbers EU544242 to EU544248).

### Satellite tracking

The two WFWDs captured at the Dagona Waterfowl sanctuary were equipped with an 18g solar powered satellite Platform Transmitter Terminal (PTT), attached on the back of the bird using a Teflon harness-attachment. Sex was determined on the basis of cloacal examination, and body mass was measured to the nearest 10 g. Before the deployment of transmitters in Nigeria a test had been conducted on captive WFWDs at Montpellier Zoo (France) to monitor the effects of attachment technique and transmitter load on birds. Movements were monitored using the Argos satellite tracking system. The PTT was programmed to transmit for a 10 h interval every 24 h according to the battery capacity. Argos CLS (Toulouse, France) processed the satellite signals and provided locations. Only locations with a precision <1000 m (location classes 1, 2 and 3, dependent on the number and quality of signals received) were used for analysis and mapping.
